# Evaluating the Response of the Soil Bacterial Community and Lettuce Growth in a Fluorine and Cadmium Co-Contaminated Yellow Soil

**DOI:** 10.3390/toxics12070459

**Published:** 2024-06-25

**Authors:** Mei Wang, Xiangxiang Chen, Yasir Hamid, Xiaoe Yang

**Affiliations:** 1 School of China Alcoholic Drinks, Luzhou Vocational and Technical College, Luzhou 646000, China; 2 Key Laboratory of Environment Remediation and Ecological Health, Ministry of Education, College of Environmental and Resource Sciences, Zhejiang University, Hangzhou 310058, China

**Keywords:** cadmium, fluorine, chlorophyll, diversity, structure, synergistic effect

## Abstract

The impact of cadmium (Cd) and fluorine (F) on plant and human health has provoked significant public concern; however, their combined effects on plant and soil bacterial communities have yet to be determined. Here, a pot experiment was conducted to evaluate the effects of exogenous F, Cd, and their combination (FCd) on lettuce growth and soil bacterial communities. The results revealed that F and Cd concentrations in lettuce ranged from 63.69 to 219.45 mg kg^−1^ and 1.85 to 33.08 mg kg^−1^, respectively, presenting lower values in shoots than in the roots. Moreover, low contamination levels had no discernable influence on lettuce growth, but showed a synergistic negative on plant biomass when exogenous F and Cd exceeds 300 and 1.0 mg kg^−1^, respectively. The results of 16S rRNA gene sequencing indicated that the most abundant bacterial community at the phylum level was *Proteobacteria*, with the relative abundance ranging from 33.42% to 44.10% across all the treatments. The contaminants had little effect on bacterial richness but impacted the structure of bacterial communities. The PCoA showed that compartment and contaminants were the primary contributors to the largest source of community variation, while the VPA indicated that F and Cd synergistically affected the bacterial communities. In turn, lettuce plants could enhance the resistance to the combined stress by increasing the relative abundance of *Oxyphotobacteria*, *Subgroup 6*, *Thermoleophilia*, and *TK10* classes in the rhizosphere.

## 1. Introduction

Heavy metals (HMs) contamination is a pressing environmental challenge globally that presents a significant threat to human health [1,2]. Among all HMs, cadmium (Cd) is a prominent pollutant, found in several regions in China [3,4]. Fluorine (F) is the 13th most abundant element in the earth’s crust and is widely recognized for its adverse effects on health in many regions around the globe [5,6]. F is often found in conjunction with Cd in the environment [7,8]. Previously, Cd was reported as a hidden toxin in an endemic fluorosis area [9]. Further, F-contaminated soil was also found in a high geochemical Cd area [10]. In addition, phosphorus fertilizer also contributes as a major source of Cd and F inputs into farmlands, and contains high concentrations of these elements, potentially increasing the concentration of Cd and F in soils and plants [6,11].

Plants exposed to F or Cd stress exhibit several negative impacts on physiological, biochemical, and morphological parameters, such as enzyme activities and metabolic processes [12,13]. Prior studies have documented the accumulation, interaction, and fractionation of Cd and F in soil-crop systems [7,14]. However, the effect on plants varied with the plant species. For instance, Chen et al. [7] reported that co-contamination of F and Cd has a synergistic negative effect on plant growth, and the contaminants are mainly accumulated in the leaves of radish (*Raphanus sativus* L.). However, it was discovered that Cd addition may restrict the F accumulation in oilseed rape (*Brassica napus* L.), while F treatment promoted the Cd accumulation in the tissues [15]. Therefore, it is vital to investigate the co-effect of F and Cd on more crop species that are closely related to human health.

Microorganisms are often more susceptible to contaminants than plants that inhabit the same soil [1,16]. Previously, numerous researches determined the effects of Cd alone or in combination with other HMs on plant growth and bacterial communities. For example, it was reported that HMs pollution has a considerable impact on the diversity, composition, and distribution of bacterial communities in soil [17]. Further, the effect of F on soil microorganisms in the soil-plant system was observed. It was found that the relative abundance of *Proteobacteria* phylum decreased with an increase in F spiking (0, 600, 1000 mg kg^−1^) in soils [18], where the F addition with 1000 mg kg^−1^ changed the bacterial diversity [19]. Moreover, the co-effects of HMs and rare earth elements (REEs), as well as the combined effects of F and Cl on soil microorganisms have garnered increasing interest. Luo et al. [16] reported that both REEs and HMs regulated the structure and composition of bacterial communities, whereas their combined impacts were higher than the individual effects of HMs or REEs. According to reports, the combination of pyrene and Cd had a greater biocidal influence on soil microorganisms than either Cd or pyrene alone [20], and the interaction between polycyclic aromatic hydrocarbons (PAHs) and HMs altered the fate of pollutants and microbial communities in soils [21]. However, to the best of our knowledge, studies on the effects of combined F and Cd on soil microorganisms are very scarce.

Lettuce (*Lactuca sativa* L.) is a globally cultivated vegetable and is commonly used for standard toxicity tests of harmful elements [22]. Previous studies have investigated the toxicity, accumulation, and translocation of co-existing metal elements in lettuce, such as Cd-Zn [23]. However, to date, the effects of combined F and Cd on lettuce plants, which may raise a potential risk to human health through food chains, have never been investigated. Considering the complex interactions of F and Cd on microorganisms and plants, the present work investigated the effects of soil spiked Cd and F on lettuce growth, soil bacterial communities, and the interactions among the pollutants, plant, and soil bacteria using a pot experiment. The main objectives of the present study were to: (1) investigate the effects of Cd or/and F on lettuce growth, and (2) explore the response of soil bacterial communities to spiked Cd or/and F in the soil-plant system. We hypothesized that: (1) there were hormetic dose—response relationships for the effect of Cd and F on lettuce growth; (2) F and Cd have a synergistic effect on plant growth and soil bacterial communities.

## 2. Materials and Methods

### 2.1. Soil Aging

The surface soil (0–20 cm) was collected from farmland in the southwest of China (105°19′26″ E, 27°22′48″ N). The basic properties of soil and analysis methods are listed in Table S1. The soil was classified as yellow soil based on its properties and the Chinese soil classification system. Before the pot experiment, the collected soil was air-dried and sieved to <2 mm. The exogenous concentrations of Cd and F in soil (Table 1) were designed according to the data of field investigation and information from previously published literature [7,15]. The soil was spiked with the appropriate amount of Cd [Cd(NO_3_)_2_∙4H_2_O] or/and F (NaF), by adding an equal volume (1.25 L) and thoroughly mixed into the soil with a total weight of 7.5 kg for each treatment. Then, the soil samples were incubated for three months at a moisture level of 60% water-holding capacity to achieve chemical equilibration in the greenhouse with the conditions were 20–25 °C, relative humidity 60–70%, and light intensity 180 μMm^−2^ s^−1^ during a 16/8 h day/night photoperiod. At the end of the aging period, 1.25 kg of soil was transferred into each plastic pot of 15 cm diameter and 12 cm height.

### 2.2. Plant Culture

Plant culture was carried out in a greenhouse at Zhejiang University. Seeds of Frisee lettuce (Huayu, Hebei, China) were bought from the local market. 20 uniform and plump seeds were evenly sown in each plastic pot. Each treatment consists of six replicates which were carried out under a completely randomized block design. The seedlings were thinned to one lettuce plant left in each plot on the 7th day of growth. The moisture content in the pots was kept at 60% water-holding capacity during the whole experimental period. The seedlings were harvested after three months of growth. Due to the limited plants and quantity of rhizosphere soils, samples collected from two replicates were combined for further analysis.

### 2.3. Plant and Soil Analysis

Chlorophyll was extracted with a mixture of acetone and ethyl alcohol and analyzed using a microplate reader (Epoch 2, BioTek, Thermo Fisher Scientific, Waltham, MA, USA) [24]. Plants were separated into roots and shoots, washed with tap water and deionized water, and then dried. The fresh weight (FW) of shoots was gauged by a balance with an accuracy of 0.001 g, and the dry weight (DW) was measured after drying at 65 °C for 72 h, while the biomass of roots remained unmeasured as certain parts of the roots remained in soils. The rhizosphere and bulk soils were collected by following the method developed by Edwards et al. [25]. Soil collected from two pots was mixed and later divided into two parts, where one was stored at −80 °C for soil DNA extraction, and the remainder was air-dried to analyze physical and chemical properties. All materials and reagents for extracting and analyzing soil microorganisms were sterilized before use.

The dried plant samples were ground and sieved to <0.43 mm, and the air-dried soil samples were passed through 2 mm and 0.15 mm sieves for analysis. The total Cd content in soil and plant was analyzed with inductively coupled plasma-mass spectrometer (ICP-MS, Agilent 7500a, Agilent Technologies Co., Ltd., Santa Clara, CA, USA) with mixed acids digestion method [24]. The analysis of F was performed using an ion-sensitive electrode method with an electrode (PF-1-01, Shanghai INESA, Shanghai, China) after samples were digested with NaOH [26]. The quality control of the measurements was performed using standard soil (GBW07405), wheat (GBW100495), and tea (GBW10016) samples bought from the Institute of Geophysical and Geochemical Exploration, China. Translocation factor (TrF) was calculated to evaluate the mobility of Cd or F from the roots to the shoots of lettuce. The bio-concentration factor (BCF) was used to assess the ability to transport and accumulate F and Cd in the lettuce plant. The equations are listed as below:

(1) $$TrF=\frac{concentration\;of\;F\;or\;Cd\;in\;shoots\;}{concentration\;of\;F\;or\;Cd\;in\;roots}$$

(2) $$BCF=\frac{concentration\;of\;F\;or\;Cd\;in\;roots}{concentration\;of\;available\;F\;or\;Cd\;in\;soils}$$

### 2.4. DNA Extraction, PCR Amplification, and 16S rRNA Gene Sequencing

Microbial DNA in soil was extracted by PowerSoil^®^ DNA Isolation Kit (MOBIO, Carlsbad, CA, USA), and quantified with Nanodrop spectrophotometer (GENE, Shanghai, China). The V3–V4 region of the bacterial 16S rRNA gene was amplified by the primer sets 338F (5′-ACTCCTACGGGAGGCAGCA-3′) and 806R (5′-GGACTACHVGG GTWTCTAAT-3′) with barcode [27]. PCR amplifications were first carried out using KOD FX Neo (TOYOBO, Japan). Briefly, the PCR reactions were amplified by thermocycling (25 cycles at 95 °C for 30 s, 50 °C for 30 s, and 72 °C for 40 s) after initialization for 5 min at 95 °C, followed by a 7 min final elongation at 72 °C. The PCR products were purified through VAHTSTM DNA Clean Beads (VAZYME, Nanjing, China). After that, the second round of PCR was performed in a 20 μL reaction which contained 10 μL 2 × Phμsion HF MM, 2.5 μL of each primer (2 μM), and 5 μL purified PCR products. Thermal cycling conditions were as follows: an initial denaturation at 98 °C for 30 s; followed by 10 cycles at 98 °C for 10 s, 65 °C for 30 s, and 72 °C for 30 s, with a final extension at 72 °C for 5 min. The PCR products was certified on 1.8% (*m*/*v*) agarose gels and quantified by the ImageJ v1.53 g software. Moreover, samples were mixed in equidensity ratios and purified with an e.Z.N.A.TM Cycle-Pure Kit (OMEGA, Connecticut, USA). The products were certified by agarose gel electrophoresis, followed by the recovery of the target fragment by using the Monarch DNA Gel Extraction Kit (GENE, Shanghai, China). Sequencing libraries were validated using a Qsep-400 Multi-Channel Bio-Fragment Analyzer (BIOPTIC, Jiangsu, China). Finally, sequencing was performed using an Illumina HiSeq 2500 platform (Illumina Inc., San Diego, CA, USA). The generated sequences were submitted in the Sequence Read Archive (SRA) database of NCBI under accession number PRJNA1101703.

### 2.5. Processing of Sequencing Data

The raw sequencing reads were primarily filtered using Trimmomatic v0.33 [28] based on the window size of 50 bp, and the reads will be cut from the start of the window once the average Q-score within the window is lower than 20. Using Cutadapt v1.9.1 [29] to identify and remove primer sequences with a maximum mismatch accepted of 20% and a minimum coverage of 80%. The paired-end reads were then assembled using Usearch v10.0 [30] followed by chimera removal using Uchime v8.1 [31]. The parameters in Usearch were set as: minimum length of overlap (10 bp), minimum similarity within overlapping regions (90%), and maximum mismatch accepted (5 bp). Chimera removal involves three steps in Uchime: (1) split query sequence into non-overlapped chunks, (2) compare these chunks with the reference database to identify the best hit of each chunk in the database and further define two best parent sequences, and (3) compare the query sequence with the two parent sequences while if a fragment with over 80% similarity to query sequence is found on both parents, this query sequence will be defined as chimera sequence. The generated sequences that had a minimum of 97% similarity were clustered into operational taxonomic units (OTUs) by using the Usearch [30], and OTUs with less than 0.005% of all sequences were removed [32]. Afterward, taxonomic annotation was processed with classify-sklearn in Qiime2 v2020.6 according to the naive Bayes classifier-based method by using SILVA132 as the reference database [33]. The classifier needs to be trained before use to “learn” which features can be used for classification, and the confidence level of the classifier was set to 0.7.

### 2.6. Statistical Analysis

One-way analysis of variance (ANOVA) based on LSD post hoc test was used to examine the variations in parameters of soil and plants among different treatments in SPSS v22.0 (IBM, Armonk, New York, USA)). Relevant figures were visualized in OriginPro 2017 (OriginLab, Northampton, MA, USA). To determine the α-diversity, the species number and Chao1 index (indicate species richness), as well as Shannon and Faith’s PD (indicate species diversity) were calculated by Qiime2. The β-diversity was analyzed in R v.4.1.1. The principal coordinate analysis (PCoA) based on the Weighted Unifrac metric was used to analyze the differences in bacterial community structures. Permutational multivariate analysis of variance (PERMANOVA) was conducted to assess the differences in community composition in R by using Vegan’s function adonis [34]. To analyze the correlation between bacterial species and soil properties, the redundancy analysis (RDA) was performed using binary Jaccard metric. Moreover, variance partitioning analysis (VPA) was used to explain the effects of F and Cd on the distribution of soil bacterial communities in R by using Vegan’s function varpart. Least discriminant analysis (LDA) based on the Kruskal—Wallis rank sum test (α = 0.05) was used to determine the species with significant differences in different treatments [35]. The relationships between the relative abundance of rhizosphere-specific bacterial taxa and parameters of soil and plants were analyzed using Pearson’s correlation test, and a heatmap was performed. Phylogenetic investigation of communities by reconstruction of unobserved states 2 (PICRUSt2) was used to predict the metagenome functional content based on the Kyoto Encyclopedia of Genes and Genomes (KEGG) pathway for function annotation [36].

## 3. Results

### 3.1. Biomass and Chlorophyll Content of Lettuce

**Figure 1:**
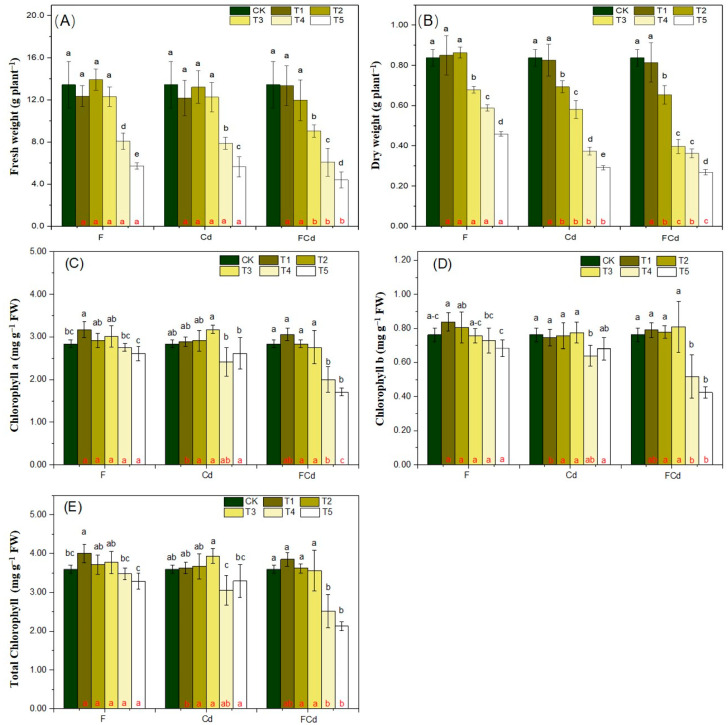
The effect of exogenous F or/and Cd in soil on fresh weight (**A**), dry weight (**B**), chlorophyll a (**C**), chlorophyll b (**D**), and total chlorophyll (**E**) of lettuce. Values are the mean ± SD (n = 3). Different black letters indicate a significant difference at *p* < 0.05 exposure to different levels of the element. Different red letters represent a significant difference at *p* < 0.05 exposure to same concentration of pollutants in Cd, F, and FCd treatments.

The effect of exogenous F or/and Cd in soil on lettuce growth was visible (Figure S1). The weight (FW and DW) of lettuce shoots showed a decreased tendency with an increasing concentration of spiked pollutants (Figure 1A,B). Compared with CK, pollutant supplements lower than 300 mg F, 1.0 mg Cd, and combined 100 mg F and 0.6 mg Cd kg^−1^ soil significantly declined the DW of lettuce shoots (*p* < 0.05, Figure 1B). Overall, synergistic negative effects of F and Cd on lettuce shoot weight were observed in the soil spiked with combined F (≥300 mg kg^−1^) and Cd (≥1.0 mg kg^−1^) compared with that of the single Cd or F treatment (Figure 1A,B).

In the case of sole F treatment, the chlorophyll a, b, and total chlorophyll concentration (mg g^−1^ FW) remained in the range of 2.60–3.17, 0.683–0.838, and 3.29–4.00, respectively (Figure 1C–E). Compared with CK, low F addition (F1) in soil increased chlorophyll concentrations, but higher F had no significant effect on chlorophyll contents (Figure 1C–E). For the Cd and FCd treatments, compared with CK, there was no statistical difference in chlorophyll concentrations with a lower rate of Cd (≤2.0 mg kg^−1^), but higher Cd addition tends to decrease the chlorophyll contents (Figure 1C–E). The negative synergy of F and Cd was detected with a higher spiking rate of F and Cd (500 and 2.0 mg kg^−1^), respectively (Figure 1C–E).

### 3.2. Concentration, Translocation and Bio-Concentration Factor of Cd and F in Lettuce

**Figure 2:**
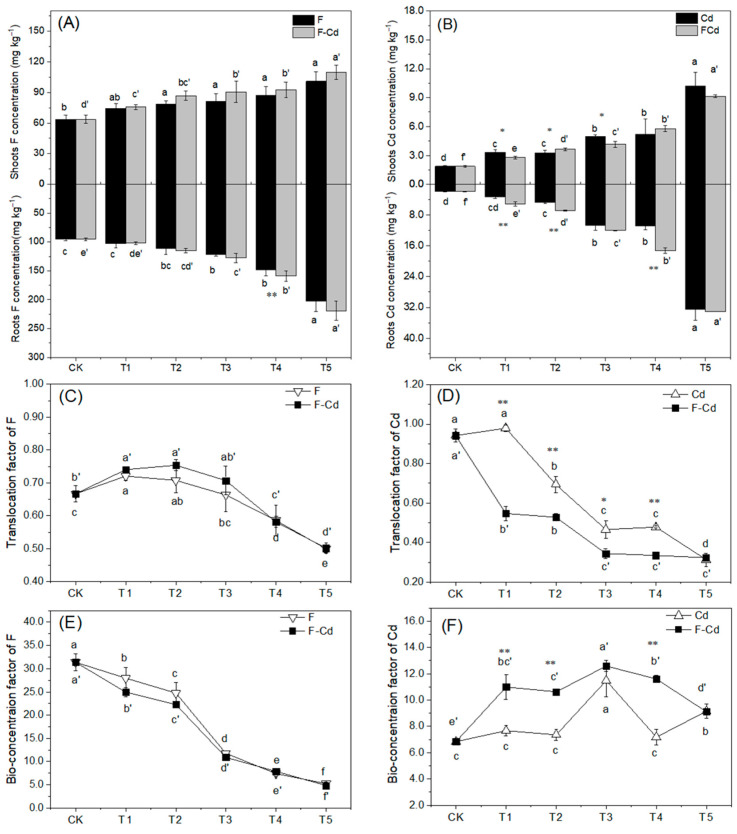
Concentration of F (**A**) and Cd (**B**) in shoots and roots of lettuce, as well as the translocation factor and bio-concentration factor of F (**C**,**E**) and Cd (**D**,**F**) in the plant. Values are the mean ± SD (n = 3). Different letters indicate a significant difference at *p* < 0.05 exposure to different levels of the element. * and ** represent a significant difference at *p* < 0.05 and *p* < 0.01, respectively, when exposure to the same level of pollutants in F, Cd, and FCd treatments.

Concentrations of Cd and F in shoots and roots of lettuce were elevated in Cd and F spiked soils (Figure 2). It was found that F concentration in the shoots (63.69–109.85 mg kg^−1^ DW) was significantly lower than that in the roots (95.47–219.45 mg kg^−1^ DW, Figure 2A). Similar trend was observed in the distribution of Cd in lettuce plants since Cd concentration in shoots and roots was in the range of 1.85–10.67 and 1.97–33.08 mg kg^−1^ DW, respectively (Figure 2B). Meanwhile, the TrF of F (0.499–0.754) first showed an increase and then tended to decrease with an increase in F in the soil (Figure 2C). A declining trend was observed for the BCF of F with values between 4.86 and 31.37 (Figure 2E). Similarly, the BCF of Cd (from 6.84 to 12.96) increased initially and then reduced in response to increasing soil Cd concentration (Figure 2F), but the TrF of Cd (0.313–0.941, except for Cd1 treatment) showed a declining trend (Figure 2D). It was observed that in the FCd treatments, the TrF of F (except for FCd4) was slightly lower than that in single F treatment (*p* > 0.5, Figure 2C); however, the TrF of Cd (except for FCd5) was often higher in Cd treatment than in FCd treatments (Figure 2D).

### 3.3. Alpha Diversity of Soil Bacteria

Across all samples, 1,893,373 high-quality clean tags were obtained, which were grouped into 1021 OTUs based on the 97% sequence similarity cutoff. The OTUs ranged from 903 to 1018, as observed in the flat rarefaction curves (Figure S2), indicating that all samples had attained saturation with OTUs. The Chao1 index ranged from 952 to 1052 with the highest richness index observed in the rhizosphere soil in FCd treatment (Table 2). The Shannon and Faith’s PD index were in the range of 7.59–7.96 and 50.46–57.30, respectively (Table 2). According to the values of OTUs, Chao1, Shannon, and Faith’s PD, the rhizosphere had higher species richness and evenness of bacteria than the bulk soil, while no obvious effect was observed for bacterial α-diversity under different pollutants (Table 2).

### 3.4. Composition of the Bacterial Community

**Figure 3:**
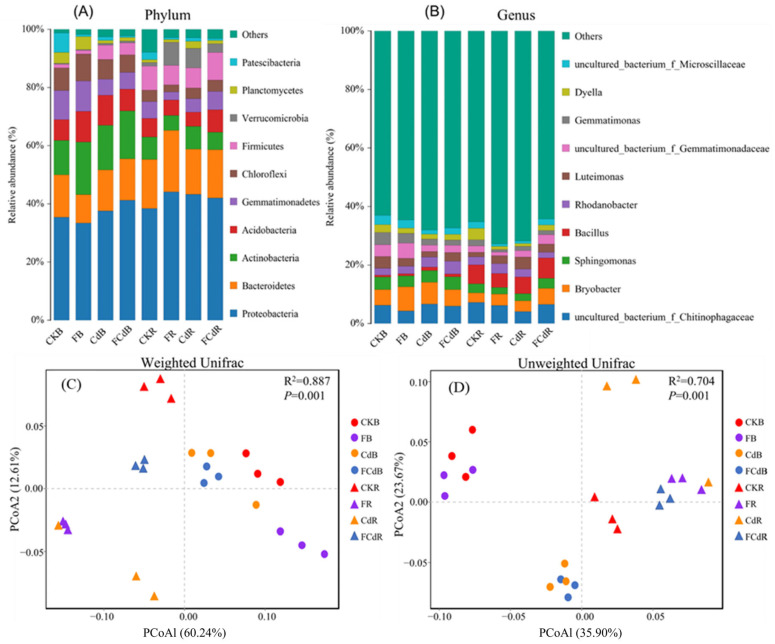
Relative abundance of dominant bacteria at the phylum level (**A**) and genus level (**B**). The principal coordinate analysis (PCoA) plots for visualization based on Weighted Unifrac (**C**) and Unweighted Unifrac (**D**) distances among the bacterial communities.

The dominant ten phyla and genera of soil bacteria were selected to generate histograms of different samples (Figure 3A,B). Bacterial sequences were primarily composed of *Proteobacteria* phylum (ranged from 33.42% to 44.10%, average of 39.45%) among all the samples (Figure 3A). The *Proteobacteria* phylum in the soil mainly consists of *Alpha-* and *Gamma-proteobacteria* with a relative abundance in the range of 17.27–25.89% and 14.85–19.80%, respectively (3B). As shown in Figure 3A, the relative abundances of *Firmicutes*, *Chloroflexi*, *Gemmatimonadetes*, and *Actinobacteria* at the phylum level varied greatly in the bulk and rhizosphere soils. As for the genus level (Figure 3B), the prominent bacteria were *uncultured_bacterium_f_Chitinophagaceae* (4.13–7.24%), *Bryobacter* (3.26–8.32%), and *Sphingomonas* (2.24–4.43%). Meanwhile, the relative abundances of *Bryobacter*, *Gemmatimonas*, *Luteimonas*, *uncultured_bacterium_f_Gemmatimonadaceae*, and *uncultured_bacterium_f_Microscillaceae* at the genus level varied greatly in the bulk and rhizosphere soils (Figure 3B).

As for bacterial community structures (the PCoA result), the first axis (>35.90%) classified the samples by compartments (rhizosphere and bulk soils) while the second axis (>12.61%) was defined by pollutants (F, Cd, and FCd) (Figure 3C,D). This was in line with the results of PERMANOVA, which showed that the compartment (41.5%, *p* < 0.01) and pollutants (26.3%, *p* < 0.05) were the primary and secondary sources of community variation, respectively (Table S2). Additionally, LDA was used to identify biomarkers with statistically significant differences between groups (Figure S3). The results showed that *Alphaproteobacteria* class, *Rhizobiales* order, *Gemmatimonadetes* phylum was the main specific bacterial taxa in lettuce rhizosphere soil under Cd, F, and FCd stress, respectively; while that in the bulk soil was phylum of *Firmicutes*, *Gemmatimonadetes*, and *Proteobacteria*, respectively. These results certified that the supplement of F, Cd, and FCd had distinct effects on the soil bacterial community.

### 3.5. Soil Properties and Pollutants Affecting the Bacterial Community

**Figure 4:**
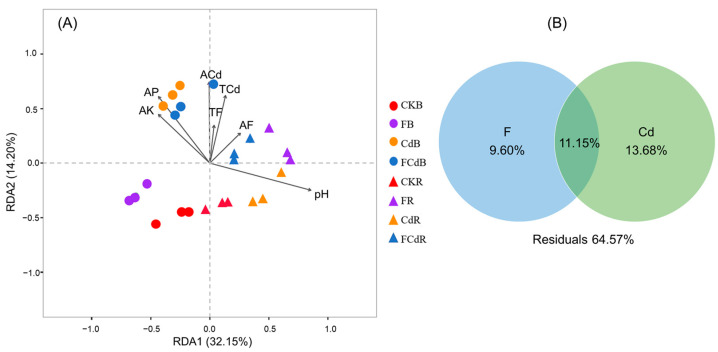
Redundancy analysis (RDA) of soil bacterial communities and selected soil properties (**A**) and variance partitioning analysis (VPA) of soil bacterial communities with soil F and Cd (**B**). AK, AP, ACd, AF represent the available phosphorus, potassium, cadmium, fluorine in soil, respectively. TCd and TF represent the total cadmium and fluorine in soil, and pH means soil pH. The magnitude and direction of correlation are shown by the length and angle of the arrows. F and Cd in the right picture represent the total and available fluorine or cadmium concentration in the soil.

The RDA showed a positive correlation between bacterial communities in the rhizosphere and the soil pH while a negative correlation was observed in bulk soil (Figure 4A). Inversely, bacterial communities in rhizosphere soil exhibited a negative correlation with soil AP and AK but positively correlated with that of bulk soil. In spite of that, F and Cd stresses (TCd, TF, ACd, AF) have no consistent effect on the bacterial community in bulk and rhizosphere soils. Soil F and Cd showed positive effects on the bacterial composition (Figure 4A). This was verified by VPA that the unique contribution of F and Cd to the variation in bacterial communities were 9.60% and 13.68%, respectively, while the pollutants’ contribution overlapped by 11.15% (Figure 4B). These results indicate that F and Cd had a synergistic effect on bacterial communities in the soil.

### 3.6. Response of Bacterial Communities to F and Cd Stresses in Rhizosphere Soil

**Figure 5:**
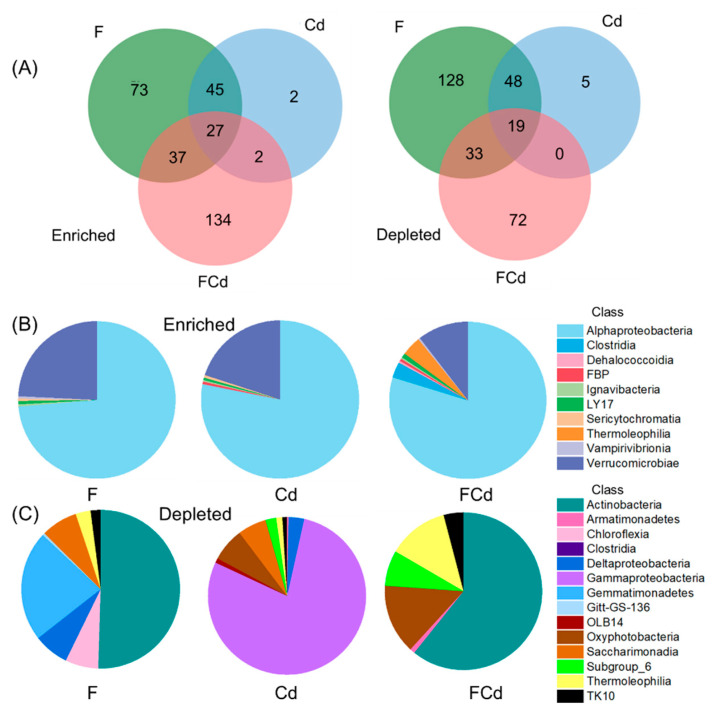
Enriched and depleted of OTUs in rhizosphere soil under different pollutants stress. (**A**) Venn diagrams displayed the enriched and depleted OTUs numbers under F, Cd, and FCd treatments (*p* < 0.05). Pie charts showed significantly enriched (**B**) and depleted (**C**) bacterial species at the family level in the rhizosphere soil under F, Cd, and FCd treatments (*p* < 0.05). Each segment in the chart is colored by the bacterial class of the corresponding taxa and the size of each segment is proportional to the relative abundance of OTUs assigned to the indicated taxa.

In comparison with CK, the rhizosphere soil of FCd treatment contained the largest number of unique enriched OTUs (134), while the lowest number of the unique depleted OTUs was 128 as observed in the rhizosphere of F treatment (Figure 5A). Further, the rhizosphere soil treated with Cd owned the least number of unique enriched OTUs (2) and depleted OTUs (5) across all treatments (Figure 5A). Among the three treatments, OTUs belonging to the *Alphaproteobacteria* class were the mainly enriched taxa in rhizosphere soil, followed by the *Verrucomicrobiae* class (Figure 5B). Compared to single F or Cd treatment, the FCd stress decreased the relative abundance of the *Verrucomicrobiae* class but increased that of the *Thermoleophilia* and *Clostridia* classes in the rhizosphere soil (Figure 5B). With regard to the depleted OTUs, there were exclusionary effects on soil bacteria, since 228, 72, and 172 OTUs were significantly depleted in the rhizosphere of F, Cd, and FCd treatment, respectively, corresponding to CK (Figure 5C). The majority of depleted OTUs in the F and FCd treatments are the *Actinobacteria* class, while those in the Cd treatment are the *Gammaproteobacteria* class (Figure 5C). Noteworthy, the FCd supplement increased the relative abundance of *Oxyphotobacteria*, *Subgroup_6*, *Thermoleophilia*, and *TK10* classes corresponding to single F or Cd stress, while that of *Saccharimonadia*, *Chloroflexia Deltaproteobacteria*, and *Gemmatimonadetes* declined in the rhizosphere soil (Figure 5C). These results indicated that the rhizosphere effects of bacteria in lettuce under F, Cd, and FCd stresses owned certain similarities but also showed apparent differences.

### 3.7. Relations of Rhizosphere-Specific Bacterial Taxa with Soil Properties and Plant Factors

**Figure 6:**
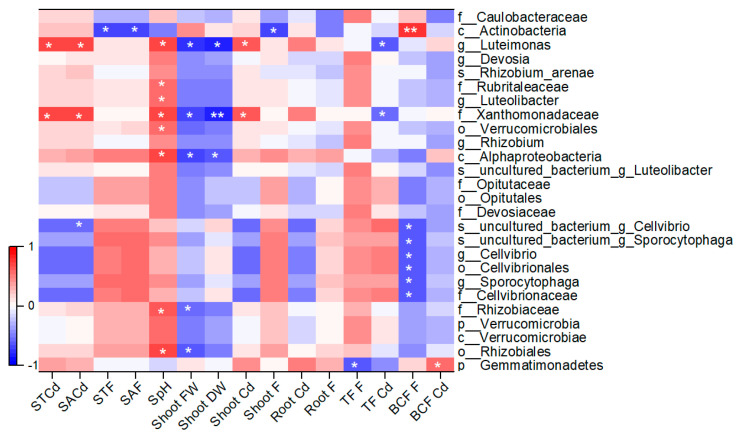
Correlations of the relative abundance of the rhizosphere-specific bacterial taxa of lettuce with soil properties and plant factors. The legend on the left side showed the range of Pearson correlation coefficients (r). The asterisks in the figure indicate significant Pearson’s correlations: ** and * represent significant at *p* < 0.01 and *p* < 0.05, respectively.

The relative abundance of rhizosphere-specific bacterial taxa was correlated with soil properties and plant factors (Figure 6). The results showed that soil pH was positively correlated with several rhizosphere-specific bacterial taxa under various stress conditions, including the *Rhizobiaceae* family and *Luteimonas* genus (*p* < 0.05). Further, soil Cd concentration positively correlated with the abundance of certain bacterial communities in the rhizosphere under single Cd stress but negatively correlated with single F stress, and vice versa (Figure 6). Moreover, the relative abundance of the *Luteimonas* genus and *Xanthomonadaceae* family positively correlated with soil Cd, pH, and shoot Cd, but they had a negative influence on shoot weight and TrF of Cd (Figure 6). The majority of enriched OTUs belonging to the *Gemmatimonadetes* phylum were negatively correlated with plant TrF of F, but positively correlated with root DW, and BCF of Cd in FCd treatment (Figure 6). These results indicate that soil, plants, and microorganisms are a unified organic combination, that interrelates and influence each other.

### 3.8. Predictive Metagenome Functional Profiling of the Bacterial Community

**Figure 7:**
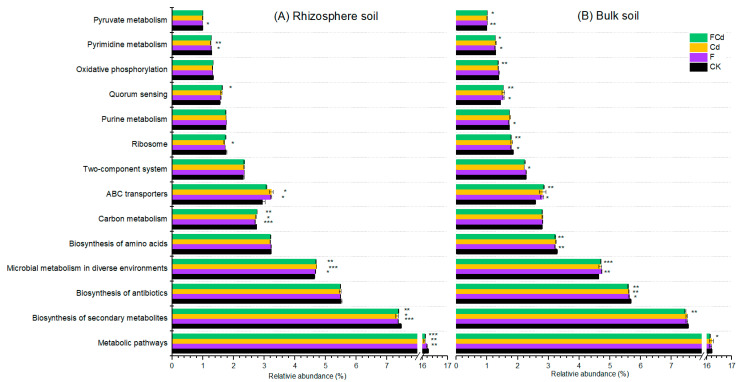
The mainly predictive functional metagenome composition in rhizosphere (**A**) and bulk soils (**B**) of lettuce under F, Cd, and FCd stresses. The *, **, and *** represent statistically significant differences at 0.05, 0.01, and 0.001 level. Only pathways with a mean proportion > 1.0% were displayed (n = 3).

Exogenous F or/and Cd affected the metabolism of bacteria in both bulk and rhizosphere soils (Figure 7), which was in accordance with the results of PERMANOVA and VPA (Table S2, Figure 4B). The KEGG-based function prediction indicated that the metabolic pathway was most important in soils, with a relative abundance greater than 16% (Figure 7). Further, biosynthesis of secondary metabolites, antibiotics, and amino acids, as well as microbial metabolism in diverse environments and ABC transporters were the main metabolic pathways with a relative abundance above 4% for all treatments (Figure 7). Notably, compared with CK, significant differences in the expression of various genes-related metabolic pathways were observed in soil polluted by F or/and Cd, indicating that bacterial communities altered several metabolic functions for environmental adaptations (Figure 7).

## 4. Discussion

### 4.1. Co-Effects of F and Cd on Lettuce Growth

To cope with abiotic stresses such as Cd and F toxicity, plants establish several strategies to mitigate stresses at molecular and physiological levels to keep them healthy in the challenging environment [37,38,39]. Further, the hormetic dose—response relationships were appropriate for Cd and F in hyperaccumulators, which indicates that low concentrations of pollutants may stimulate plant growth but excessive exposure may lead to toxicity [40]. Previously, lettuce has been reported as a species with a high capacity for Cd accumulation and growth stimulation in the presence of low Cd concentrations [41]. While F is the 13th most abundant and highly electronegative element on the earth, it is abundantly and ubiquitously distributed in the environment [42]. Therefore, low supplementation of F (<300 mg kg^−1^) or/and Cd (<1.0 mg kg^−1^) to the soil had no visible effect on lettuce growth as found in the present study (Figure 1).

In spite of that, plants have evolved a well-developed immune system to mitigate abiotic stresses [38,39], it remains difficult to protect lettuce plants from the detrimental effects of excess F or/and Cd stress (Figure 1). It is well established that exposure to F or Cd stress could induce leaf chlorosis thereby reducing plant photosynthesis [37]. In this study, high levels of F or Cd in soil inhibited lettuce growth, and decreased the FW and DW, as well as the chlorophyll contents (Figure 1) which were in line with previous reports [43]. Further, compared with the single F or Cd treatments, the plant weight and chlorophyll contents in FCd treatments (especially in FCd4,5) were decreased which coincides with previous studies on radish and oilseed rape plants [7,15]. In sum, these results confirmed the synergistic effect of F and Cd on the growth of lettuce.

Moreover, the differences in absorption and transportation pathways of Cd and F and their action sites in lettuce plants might explain their negative synergistic effect. The uptake of F from soil into plant roots mainly occurs through a passive diffusion process [44] as well as a partial active process via ion channels, e.g., Cl^−^ channel [45]. As for Cd, both passive and active transports have been reported for Cd transportation in the plasma membrane of root cells, with the latter requiring the usage of divalent cations, such as Ca^2+^, Fe^2+^, and Zn^2+^ [37]. Further, Cd and F ions in solution could co-exist as CdF^+^, which could be absorbed by plants [15]. Moreover, F^−^ can react with Ca^2+^ to generate precipitation of CaF_2_ in the solution [12] which could decrease the competition of Ca^2+^ and Cd^2+^ at the surface of roots. These complicated processes consequences in the higher uptake of F and Cd by lettuce roots in FCd treatment than in the single F or Cd treatment. This was consistent with the result of the present study that the concentration and BCF of F and Cd in FCd treatment were repeatedly higher than that in the single F or Cd treatment (Figure 2).

The result in this study was partially similar to Chen et al. [7] that adding Cd into F treatments weakened the F transportation from root to shoot, while adding F into Cd treatments had no discernible effect on the TrF of Cd in radish plants (Figure 2C,D). Although the translocation of F and Cd from root to shoot was unclear, the different translocation properties of the two elements were observed. Apoplastic transport was involved in F transportation [45], and the absorbed F was transported from roots to shoots via the transpiration stream [46]. However, both apoplastic and symplastic pathways were involved in the transport of Cd [37]. It was also reported that a long-distance translocation of Cd may be facilitated by transpiration but can be determined by several factors, such as the amount of Cd sequestered in the vacuole and the availability of other elements [47]. Previously, the translocation of metal—F complexes, such as Al-F, Pb-F, Cd-F, and As-F through the apoplast or via dedicated membrane-bound xenobiotic transporters were reported [47]. In summary, the transport of F and Cd in plants have some independent processes, but the two elements also interact with each other to some extent, and this complicated and changeable system was affected by various ions. As a result, the biochemical and physiological properties of F and Cd as well as the response of the lettuce plant to the stresses induced the synergistic negative effect when F and Cd addition in the yellow soil exceeds 300 and 1.0 mg kg^−1^, respectively. Considering the concentration of Cd (0.52 mg kg^−1^) and F (925 mg kg^−1^) in the un-spiked soil (Table S1), the total Cd and F concentration in the soil was higher than 1.52 and 1225 mg kg^−1^, respectively. According to the risk screening values for soil contamination of agricultural land in the State Standard of the People’s Republic of China and the evaluation method used in the National soil pollution survey bulletin in China in 2014, the soil was moderately contaminated with Cd. Moreover, F concentration also greatly varies with soil type, thus no specified standard has set the limit value of F in soils in China. However, according to the survey by China National Environmental Monitoring Center, the background value of F in Chinese topsoil was 453 mg kg^−1^ with a range of 191 to 1012 mg kg^−1^. The soil with an F concentration of higher than 1225 mg kg^−1^ in the present study was polluted. It is worth noting that the high total soil F concentrations were commonly found in endemic fluorosis areas in southwest China [6]. High F and Cd contents in soil not only impair plant growth, but also endanger human health through the food chain and disrupt ecological balance. Moreover, Cd and F in the human body have analogous exposure sources, similar target organs of the toxicities, and analogical clinical symptoms in teeth and bones [48]. Therefore, the migration and transformation of soil Cd and F along the food chain and the health risk assessment of human beings should be directed in future studies.

### 4.2. Response of the Soil Bacterial Community to F and Cd Stress

It is well established that geographical location, soil types, plant species, cultivation practices, soil compartments, and contaminants pose effects on bacterial communities [25,27]. In this study, the impact of F and Cd on the soil bacterial community was investigated in a soil-plant system. As reported in previous reports [19,27], the rhizosphere and bulk soils were dominated by *Proteobacteria*, *Bacteroidetes,* and *Actinobacteria* (Figure 3). Further, the compartment analysis explained the largest source of variation (Figure 3 and Figure 4; Table S2), indicating the importance of niche filtering that soil microorganisms and lettuce roots may recruit specific microbiota from the bulk soil, which subsequently colonizes in the rhizosphere soil of lettuce. As for the genus level, *Luteimonas* accounted for 1.62–4.14% abundance in this study (Figure 3B) and was positively correlated with the total and available Cd content in the soil (Figure 6). It indicated that *Luteimonas* was Cd tolerant.

Despite the lack of a clear relationship between the bacterial diversity of soil spiked with various pollutants and its bacterial community, it is worth noting that F and Cd were also critical factors and acted synergistically on the soil bacterial community (Figure 4). This was in line with the result that microbial diversity was not significantly affected by HMs contamination, but their composition was significantly affected by the pollution [49]. A similar trend was observed by Luo et al. [16] that HMs and REEs affected the bacterial communities and their combined contributions were greater than the single effects. Organisms can enrich and accumulate F and Cd simultaneously [7,15], as well as high F and Cd concentrations significantly inhibit the microorganisms [19,50]. Therefore, F and Cd probably have a synergistic effect on bacterial communities in soil.

The Rhizosphere acts as an active region for the nutrient and energy exchange in the soil-plant system, and is referred to as a vital barrier for pollutants uptake. Moreover, it is also considered as an important region for pollutant—microorganism interactions [16]. Therefore, the response of bacterial taxa to F and Cd stress, and the interactions of plant-bacteria and soil-bacteria systems were discussed. In our study, compared with the CK, rhizosphere soils spiked with F, Cd, and FCd primarily enriched OTUs belonging to the *Alphaproteobacteria* class (Figure 4B), which might be due to the ecological and biochemical ability to degrade contaminants [51]. Further, OTUs belonging to *Verrucomicrobiae* class were enriched in the polluted rhizosphere soil corresponding to CK (Figure 4B), while *Verrucomicrobia* phylum was enriched in lettuce rhizosphere corresponding to the bulk soil (Figure 3A). Similarly, enrichment of *Verrucomicrobia* in the rhizosphere of maize was reported which might relate to the maintenance of bacterial populations homeostasis in the rhizosphere, since it can form beneficial interactions with plant roots [52]. Furthermore, the enriched OTUs in the rhizosphere under FCd stress were higher than single F or Cd treatment (Figure 4B,C) indicating that the microbial community shifted to strengthen the adaptability of microorganisms towards the pollutants [49]. Interestingly, compared with CK, OTUs belonging to the *Clostridia* class were enriched in the rhizosphere of FCd treatment, but depleted in single F and Cd treatments (Figure 4B,C). *Clostridia* are often described as anaerobic organisms, thus the porosity and permeability of the rhizosphere soil, which is intimately connected to plant roots was improved compared with the bulk soil, especially in yellow clay soil (Table S1). In the *Clostridia* class, multiple plant-pathogenic *Clostridium* species could cause soft rot disease on plants [53], implying that lettuce plants under combined FCd stress have a higher probability of suffering the disease as compared with plants under single F or Cd stress.

*Actinobacteria* perform a variety of functions in soil-plant systems such as decomposing soil organic matter, elevation in nutrient absorption, disease prevention and suppression in plants, and alleviating plant biotic and abiotic stresses [54]. Compared with CK, OTUs belonging to the *Actinobacteria* class were depleted in the rhizosphere of lettuce in F and FCd treatments but remained unaffected by single Cd stress (*p* > 0.05, Figure 4C), indicating that these taxa were more sensitive to F than Cd stress. However, the *Gammaproteobacteria* class was susceptible to Cd than F stress as these OTUs were significantly depleted in the rhizosphere under Cd stress (*p* < 0.05) with no obvious difference in F and FCd treatments (Figure 4C). Our results were following the findings of Gan et al. [19] who reported no clear relationship between *Gammaproteobacteria* abundance and F content in soil. On the other hand, the synergistic effect of F and Cd on the depleted OTUs belonging to *Saccharimonadia*, *Chloroflexia, Deltaproteobacteria*, and *Gemmatimonadetes* classes in lettuce rhizosphere (Figure 4C) may contribute to the discrepancy of bacterial communities influenced by F and Cd (Figure 5B). Plants can select specific soil bacterial consortia [55]; therefore, these depleted OTUs which are beneficial for substance circulation in soil and nutrient uptake in plants might participate in inhibiting the growth of lettuce.

Earlier research has confirmed that elevated levels of pollutants in soil might be toxic to a variety of microorganisms due to their effects on metabolic functions [16]. Consistent with a report, bacteria associated with metabolism pathways are the most abundant components in soils [56]. Moreover, in this study, Cd or/and F stresses resulted in a decrease in the relative abundance of genes associated with the degradation and utilization of organic matter (such as carbon metabolism). However, it led to an increase in the genes related to the resistance and transportation of pollutants (such as ABC transporters) (Figure 7). However, due to the limitations of PICRUSt, using multi-omics techniques including metagenomics and transcriptomics to unravel the functional categories responsible for the co-effects of Cd and F in plants is necessary in future research.

## 5. Conclusions

This study examined the impact of F or/and Cd pollutions in soil on the response of the soil bacterial community and lettuce growth under a pot trial. The results indicated that the concentration of Cd and F in the lettuce plant increased with elevated F and Cd levels in soils, with values ranging from 63.69–219.45 and 1.85–33.08 mg kg^−1^, respectively. Low concentrations of F and Cd have no obvious effect on plant weight and chlorophyll contents, but excess F and Cd declined these indexes resulting in reduced lettuce growth. Further, soil bacterial diversity was not visibly affected by F or/and Cd; however, the community composition was altered under the stresses. Detailed analysis revealed that compartments were the primary factor contributing to the greatest amount of variation within the community, followed by F and Cd pollution which might have a synergistic effect on the soil bacterial community. In the rhizosphere of lettuce, to alleviate F or/and Cd stresses, bacteria associated with the resistance and transport of pollutants were increased. Future studies should focus on the mechanisms of the synergistic effect of F and Cd on plants and microorganisms, as well as their combined effects on human health by using multi-omics technologies, such as genomics, transcriptomics, proteomics, and molecular biology.

## Figures and Tables

**Table 1:** Exogenous concentrations of F and Cd in soil. Treatment included with F (F1 to F5), Cd (Cd1 to Cd5), and FCd (FCd1 to FCd5). A soil without addition was conducted as control treatment (CK).

Treatment	F mg kg^−1^	Cd mg kg^−1^	Treatment	F mg kg^−1^	Cd mg kg^−1^	Treatment	F mg kg^−1^	Cd mg kg^−1^
CK	0	0						
F1	50	0	Cd1	0	0.3	FCd1	50	0.3
F2	100	0	Cd2	0	0.6	FCd2	100	0.6
F3	300	0	Cd3	0	1.0	FCd3	300	1.0
F4	500	0	Cd4	0	2.0	FCd4	500	2.0
F5	1000	0	Cd5	0	5.0	FCd5	1000	5.0

**Table 2:** Summary of alpha diversity metrics. Values are the mean ± SD (n = 3). CKB, CdB, FB, and FCdB represent soil samples collected from bulk soil in treatment of CK, Cd4, F4, and FCd4, respectively. CKR, CdR, FR, and FCdR represent soil samples collected from rhizosphere soil in treatment of CK, Cd4, F4, and FCd4, respectively. Different letters represent a significant difference at *p* < 0.05 exposure different treatments. The same as below.

Sample ID	OTUs	Chao1	Shannon	Faith’s PD
CKB	915.00 ± 2.65 c	962.82 ± 5.34 c	7.66 ± 0.03 bc	50.97 ± 0.48 c
FB	902.67 ± 4.93 c	952.34 ± 9.00 c	7.59 ± 0.10 c	50.46 ± 0.46 c
CdB	965.67 ± 19.30 b	1001.66 ± 14.17 b	7.79 ± 0.02 abc	54.40 ± 0.31 b
FCdB	967.67 ± 14.15 b	999.09 ± 29.13 b	7.82 ± 0.04 abc	54.15 ± 0.26 b
CKR	1014.00 ± 36.76 a	1040.26 ± 31.67 a	7.73 ± 0.35 abc	56.91 ± 1.86 a
FR	1009.67 ± 3.06 a	1041.43 ± 2.69 a	7.79 ± 0.02 abc	57.36 ± 0.19 a
CdR	974.33 ± 13.50 b	1029.03 ± 6.10 ab	7.90 ± 0.06 ab	54.86 ± 1.70 b
FCdR	1018.00 ± 8.19 a	1051.79 ± 18.44 a	7.96 ± 0.03 a	57.30 ± 0.49 a

## Data Availability

The original contributions presented in this study are included in this article, and further inquiries can be directed to the corresponding authors.

## Supplementary Materials

The following supporting information can be downloaded at: https://www.mdpi.com/article/10.3390/toxics12070459/s1, Table S1: Physicochemical properties of the tested soil; Table S2: Determining the environmental variables affecting the microbial community composition using PERMANOVA; Figure S1: Photo of lettuce (*Lactuca sativa* L.) at harvest; Figure S2: The rarefaction curves of samples; Figure S3: Line discriminant analysis (LDA) value distribution histogram for bacterial taxa in rhizosphere soil and bulk soil of lettuce.
