# Supplementary material for: Evaluating the Response of the Soil Bacterial Community and Lettuce Growth in a Fluorine and Cadmium Co-Contaminated Yellow Soil
Source: Toxics. 2024 Jun 25;12(7):459. doi: 10.3390/toxics12070459 (PMC11280846; doi:10.3390/toxics12070459)
Supplement: Supplementary file 1 [file toxics-12-00459-s001.zip › toxics-3044998-supplementary.pdf]

toxics

MDPI

*Supplementary Materials*

# Evaluating the Response of the Soil Bacterial Community and Lettuce Growth in a Fluorine and Cadmium Co-Contaminated Yellow Soil

**Mei Wang [1,2,*], Xiangxiang Chen [1], Yasir Hamid [2] and Xiaoe Yang [2,*]**

[1] School of China Alcoholic Drinks, Luzhou Vocational and Technical College, Luzhou 646000, China
[2] Key Laboratory of Environment Remediation and Ecological Health, Ministry of Education, College of Environmental and Resource Sciences, Zhejiang University, Hangzhou 310058, China
* Correspondence: lzywangmei@163.com (M.W.); xeyang@zju.edu.cn (X.Y.); Tel.: +86-13551108496 (M.W.); +86-13858085377 (X.Y.)

**Table S1.** Physicochemical properties of the tested soil.

| Parameters | Values | Methods or instruments |
|---|---|---|
| pH (1:2.5) | 6.01±0.04 | pH detector (NY/T 1121.2-2006) |
| OM (g kg–1) | 35.37±0.01 | Walkley-Black wet digestion method (Bao, 2008) |
| AvN (g kg–1) | 1.03±0.00 | Diffusion method (Bao, 2008) |
| AvK (g kg–1) | 2.48±0.03 | CH3COONH4 extraction (NY/T 89-2004) |
| AvP (mg kg–1) | 94.89±6.67 | NH4F-HCl extraction (NY/T 1121.7-2014) |
| TCd (mg kg–1) | 0.52±0.03 | HNO3-HF-HClO4 digestion, ICP-MS (7500a, Agilent) |
| TF (mg kg–1) | 925.12±33.19 | NaOH digestion, ion-selective electrometry method |
| AvCd (mg kg–1) | 0.29±0.00 | DTPA extraction, ICP-MS (7500a, Agilent) |
| AvF (mg kg–1) | 3.05±0.10 | Water extraction, ion-selective electrometry method |
| soil texture: sand (%) | 13.36%±0.45% | |
| silt (%) | 19.64%±1.30% | Hydrometer method (NY/T 1121.3-2006) |
| clay (%) | 67.01%±0.85% | |

NY/T 1121.2-2006 Soil Testing Part 2: Method for determination of soil pH. The Ministry of Agriculture of the People's Republic of China. Beijing, China.

NY/T 1121.7-2014 Soil Testing Part 7: Method for determination of available phosphorus in soil. The Ministry of Agriculture of the People's Republic of China. Beijing, China.

NY/T 89-2004. Determination of exchangeable potassium and non-exchangeable potassium content in soil. The Ministry of Agriculture of the People's Republic of China. Beijing, China.

NY/T 1121.3-2006 established by Ministry of Environmental Protection and by Ministry of Agriculture of the People's Republic of China

Bao, S.D. Soil agricultural chemistry analysis method, 3rd edn. China Agriculture Press, Beijing. 2008, pp.34–35.(in Chinese)

**Table S2.** Determining the environmental variables affecting the microbial community composition using PERMANOVA.

| Environmental variables | Bray Curtis | | Binary Jaccard | | Weighted Unifrac | | Unweighted Unifrac | |
|---|---|---|---|---|---|---|---|---|
| | R2 | P value | R2 | P value | R2 | P value | R2 | P value |
| Compartment | 0.415 | 0.001 | 0.302 | 0.001 | 0.480 | 0.001 | 0.321 | 0.001 |
| Pollutant | 0.263 | 0.025 | 0.204 | 0.055 | 0.194 | 0.136 | 0.200 | 0.074 |

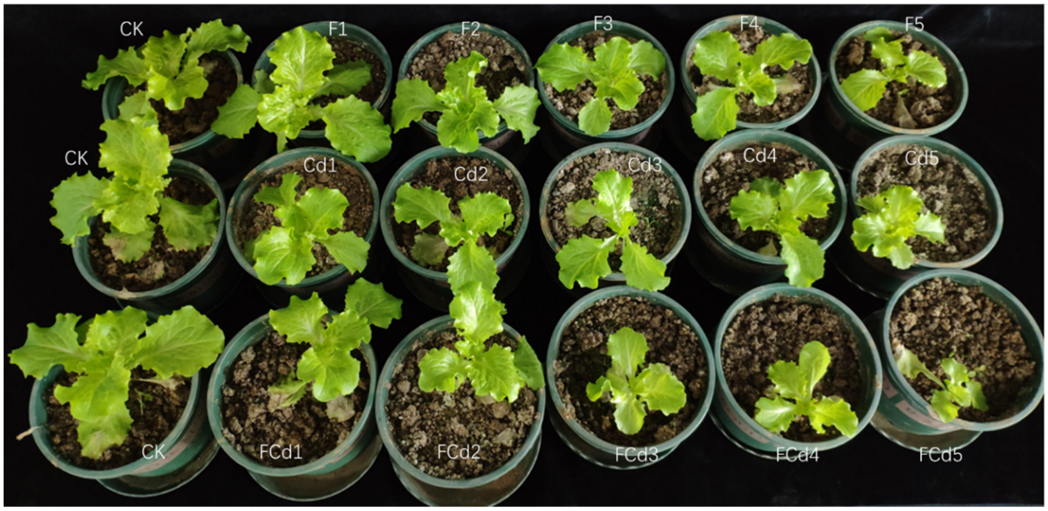


**Figure S1.** Photo of lettuce (Lactuca sativa L.) at harvest. F1 to F5 represent treatment at the exogenous concentration of fluoride in soil was 50, 100, 300, 500, and 1000 mg kg–1, respectively. Cd1 to Cd5 represent treatment at the exogenous concentration of cadmium in soil was 0.3, 0.6, 1.0, 2.0 and 5.0 mg kg–1, respectively. FCd1 to FCd5 represent soil added with combined fluoride and cadmium at the same level of F1-Cd1 to F5-Cd5.

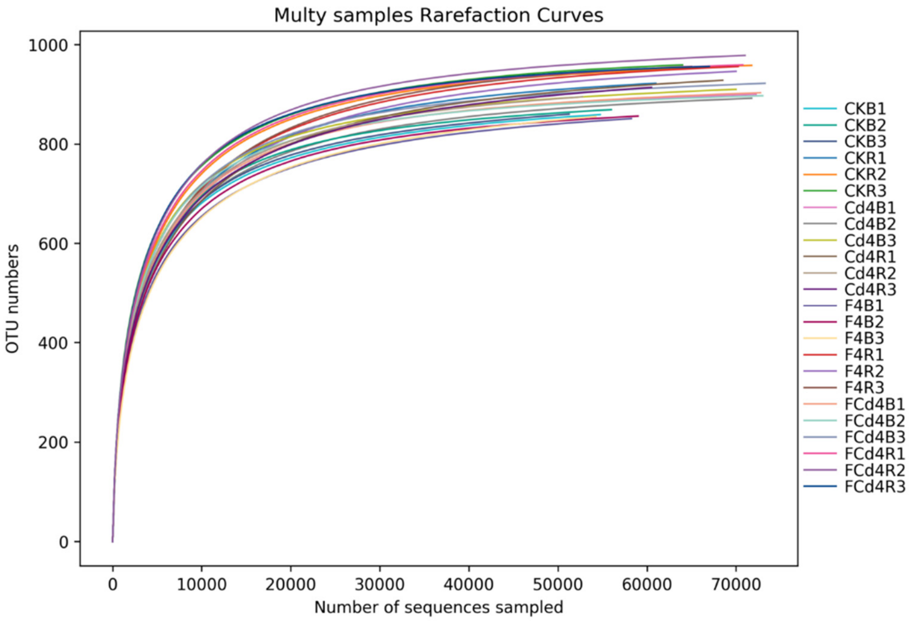


**Figure S2.** The rarefaction curves of samples. CKR, Cd4R, F4R, and FCd4R represent soil samples collected from rhizosphere soil for treatment of CK, Cd4, F4, and FCd4, respectively. CKB, Cd4B, F4B, and FCd4B represent soil samples collected from bulk soil for treatment of CK, Cd4, F4, and FCd4, respectively. Each treatment was carried out in triplicate.

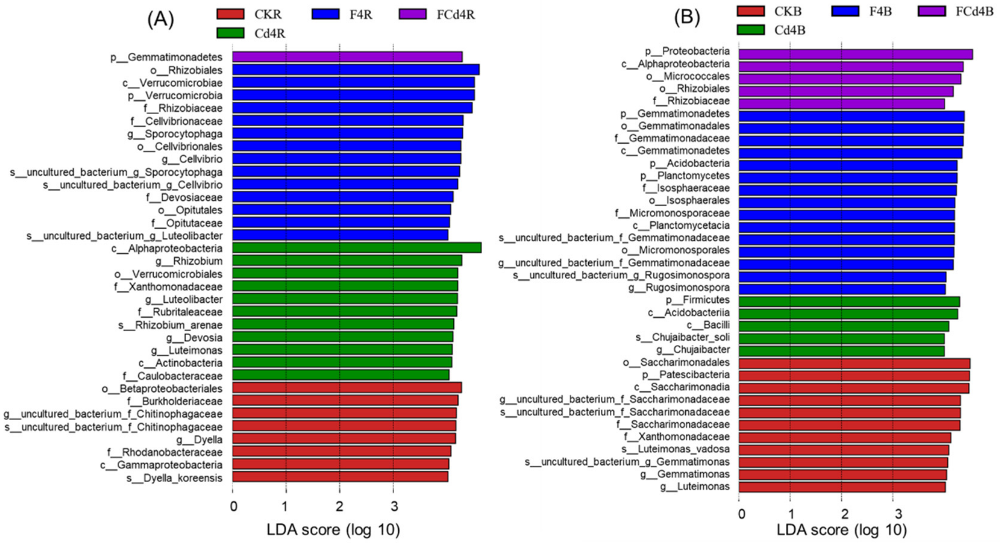


**Figure S3.** Line discriminant analysis (LDA) value distribution histogram for bacterial taxa in rhizosphere soil (A) and bulk soil (B) of lettuce under F, Cd, and FCd stress. The figure showed the species that LDA Score is higher than the default value of 4.0. CKR, Cd4R, F4R, and FCd4R represent soil samples collected from rhizosphere soil for treatment of CK, Cd4, F4, and FCd4, respectively; CKB, Cd4B, F4B, and FCd4B represent soil samples collected from bulk soil for treatment of CK, Cd4, F4, and FCd4, respectively.
